# University Dropout: A Systematic Review of the Main Determinant Factors (2020-2024)

**DOI:** 10.12688/f1000research.154263.2

**Published:** 2024-11-12

**Authors:** Raul Quincho Apumayta, Javier Carrillo Cayllahua, Abraham Ccencho Pari, Vilma Inga Choque, Juan Carlos Cárdenas Valverde, Delsio Huamán Ataypoma

**Affiliations:** 1 School of Social Sciences and Rural Development, Faculty of Education Sciences, National University of Huancavelica, Avenida Agricultura 319-321, Paturpampa, Huancavelica, 09001, Peru; 2 School of Early Childhood and Bilingual Intercultural Education, Faculty of Education Sciences, National University of Huancavelica, Avenida Agricultura 319-321, Paturpampa, Huancavelica, 09001, Peru; 3 School of Agro-industrial Engineering, Autonomous High Andean National University of Tarma, La Florida – Cochayoc Highway, km 2, Huancucro N° 2092, Tarma - Junin, 120701, Peru

**Keywords:** University dropout, vocational guidance, academic performance, socioeconomic status, institutional aspect

## Abstract

**Introduction:**

This research is a systematic review aimed at synthesizing scientific evidence on the causes of university dropout, focusing on the subcategories of vocational guidance, academic performance, socioeconomic status, and institutional aspects between 2020 and June 2024.

**Methods:**

Only articles addressing university dropout were considered, analyzing dimensions such as vocational guidance, academic performance, socioeconomic status, and institutional aspects. Articles published in indexed scientific journals with double-blind, double-blind peer, or open reviews between 2020 and June 2024 were included. The main databases used were Scopus, Web of Science, and Google Scholar. To assess the risk of bias in qualitative studies, the criteria from the article “Validity criteria for qualitative research: three epistemological strands for the same purpose” were used. For quantitative studies, the criteria from the article “Evaluating survey research in articles published in Library Science journals” were followed. For mixed-method studies, both sets of criteria were combined.

**Results:**

A total of 23 studies were included: 15 quantitative (65.22%), 3 qualitative (13.04%), and 5 mixed-method (21.74%). All studies (100%) addressed the subcategories of socioeconomic status and institutional aspects. Regarding the academic performance subcategory, 86% of the studies addressed it, while the vocational guidance subcategory was covered by 73.91% of the studies.

**Conclusions:**

Vocational guidance, academic performance, socioeconomic status, and institutional aspects are crucial for reducing university dropout. Providing adequate professional guidance, academic support, financial assistance, and strong institutional support is fundamental to improving student retention and academic success.

## Introduction

University dropout is a multifaceted problem that affects both educational institutions and society at large. This phenomenon is influenced by several key factors. Inadequate vocational guidance can lead to poor career choices, resulting in a disconnection from studies and, ultimately, dropout. Providing proper career guidance before the start of studies allows for informed decisions, reducing the risk of dropout. Academic performance also plays a crucial role, as students with better grades are less likely to abandon their studies. However, academic performance alone is not sufficient to ensure retention if it is not addressed alongside other factors ( Bernardo et al., 2017; Chalela-Naffah et al., 2020; Kim & Kim, 2018; Santos-Villalba et al., 2023; Schmidt et al., 2023).

Socioeconomic status is another critical determinant, as financial difficulties can force students to abandon their studies to work or attend to family responsibilities. Finally, institutional aspects, such as satisfaction with study programs, integration into the academic environment, and institutional support, are fundamental for reducing dropout rates. An integrated approach that considers vocational guidance, academic performance, socioeconomic status, and institutional aspects is essential to effectively address university dropout ( Chalela-Naffah et al., 2020; Kim & Kim, 2018; Schmidt et al., 2023). Additionally, other important reasons include lack of motivation, low self-esteem, and frustration ( dos Santos et al., 2022; Garcés-Delgado et al., 2024; Garcés-Prettel et al., 2024).

The impact of university dropout extends to economic, personal, and institutional levels. Dropout leads to frustrated professionals, a lower intellectual and productive contribution to society, and economic costs for families and institutions. Additionally, dropout is associated with difficulties in adapting to university studies, personal and professional challenges, and a lack of emotional support ( Garcés-Prettel et al., 2024).

To prevent university dropout, it is essential to constantly monitor academic performance and include formal academic reinforcement activities ( Santos-Villalba et al., 2023). Educational policies should be designed with the realities and needs of students in mind, using innovative methodologies to increase motivation ( Garcés-Prettel et al., 2024; Kim & Kim, 2018). Communication between university administration, faculty, and students is crucial in academic retention processes ( Chalela-Naffah et al., 2020; Valencia-Arias et al., 2023).

The economic implications of university dropout are significant. Dropout represents a high percentage of public expenditure on higher education and has economic consequences for families and institutions ( Santos-Villalba et al., 2023). Socioeconomic factors and the income score provided by the University Admission Exam are important variables influencing student dropout ( Viloria et al., 2019). The cumulative effect of low admission scores and living far from family support results in a higher probability of dropout, highlighting the economic impact on students ( Chalela-Naffah et al., 2020).

Likewise, university dropout is influenced by a multitude of factors, including academic performance, economic difficulties, and dissatisfaction with the educational experience. The impact of dropout on students includes personal and professional challenges, while the economic implications are significant for both individuals and institutions. Strategies to prevent dropout involve constant monitoring of academic performance, designing educational policies aligned with student needs, and implementing interventions that provide support and motivation to at-risk students.

The relevance of studying the category of university dropout lies in a comprehensive understanding of its subcategories. In terms of inadequate vocational guidance, it contributes to early dropout, as many students enter programs that do not align with their interests or abilities, increasing the likelihood of withdrawal ( Lorenzo-Quiles et al., 2023). Low academic performance, especially in the first year, is also a critical factor, impacting approximately 20-30% of students in some countries ( OECD, 2019). Additionally, socioeconomic status has a decisive influence, as students from lower socioeconomic backgrounds face greater challenges in covering educational costs, which results in inefficient use of resources such as scholarships and financial aid intended to prevent dropout ( Mtshweni, 2021). Finally, institutional aspects, such as the quality of support services and the availability of academic and social resources, are also critical factors affecting retention. Institutions with deficiencies in these areas experience a higher dropout rate, impacting both the academic structure and the financial resources available ( Guzmán Rincón et al., 2021).

All the theoretical information addressed so far leads to the following question: What scientific evidence exists regarding the causes of university dropout related to vocational guidance, academic performance, socioeconomic status, and institutional aspects between 2020 and June 2024? The general objective is to systematize the scientific evidence on the causes of university dropout, focusing on the subcategories of vocational guidance, academic performance, socioeconomic status, and institutional aspects between 2020 and June 2024.

## Methods

For the development of this research, the PRISMA 2020 methodology was essential, providing a comprehensive and rigorous framework for conducting a systematic review of university dropout ( Page et al., 2021). Its 27-item checklist facilitated the selection, synthesis, and reporting of studies, ensuring transparency and minimizing bias in the findings ( Page et al., 2021). Additionally, its integration of advanced methodologies, such as the use of digital tools for analysis, strengthened the consistency and reproducibility of results, which are crucial in the educational field ( Brennan & Munn, 2021). This approach enabled a structured and reliable review, aligned with educational research needs.

### Inclusion criteria

International studies published between 2020 and June 2024 were included, focusing on the factors of university dropout. The studies had to employ qualitative, quantitative, and mixed methodological approaches, presenting a standardized methodological design. Only articles addressing the category of university dropout were considered, analyzing at least two of the subcategories of vocational guidance, academic performance, socioeconomic status, and institutional aspects. These studies could come from public or private universities or other higher education institutions. The articles needed to be published in indexed scientific (in any language) journals with double-blind, double-peer blind, or open reviews.

### Exclusion criteria

Studies published outside the period from 2020 to June 2024, documents that were not research articles, and those that did not specifically focus on university dropout or did not consider at least 50% of the study subcategories were excluded. Studies with poor methodological design were also discarded ( Table 2).

Table 1 shows a total of 23 investigations, distributed as follows: 15 quantitative studies (65.22%), 3 qualitative studies (13.04%), and 5 mixed-method studies (21.74%). All studies (100%) addressed the subcategories of socioeconomic status and institutional aspect. The subcategory of academic performance was covered by 86% of the studies, while the subcategory of vocational guidance was addressed by 73.91% of the studies. The table also includes relevant general methodological information for each included study. Of all the studies included, spanning 12 different countries, the largest concentration was in Colombia with 5 studies (21.74%), followed by Spain with 4 (17.39%) and Mexico with 3 (13.04%). Germany and Chile each contributed two studies (8.70%), while Denmark, Peru, Ecuador, South Africa, the Dominican Republic, Kosovo, and Cuba each contributed one study (4.35%).

**Table 1.** General characteristics of included studies.

No.	Authors, Year	Country	Category (University Dropout)				General Methodological Information
			1	2	3	4	
1	( Thies & Falk, 2024)	Germany	X	X	X	X	Approach: Quantitative Design: Longitudinal Population and Sample: International students who began their studies at German universities in the winter semester of 2017-2018 Sample: 3660 students Techniques and Instruments: Online survey
2	( Thomsen, 2022)	Denmark	-	X	X	X	Approach: Quantitative Design: Longitudinal Population and Sample: Students enrolled for the first time in a university bachelor’s program in Denmark between 1993 and 2006 Sample: 185,939 students Techniques and Instruments: Document review
3	( Geisler et al., 2023)	Germany	-	X	-	X	Approach: Quantitative Design: Longitudinal Population and Sample: First-year students enrolled in a pure mathematics program or a mathematics teacher training program at a public university in Germany Sample: 274 students Techniques and Instruments: Questionnaire
4	( Gonzales Lopez & Evaristo Chiyong, 2021)	Peru	-	X	X	X	Approach: Quantitative Design: Quasi-experimental Population and Sample: First-year university students enrolled in a Descriptive Statistics and Probabilities course at a private university in Lima (39 in the control group and 28 in the experimental group) Techniques and Instruments: Measurement scale
5	( Pertegal-Felices et al., 2022)	Ecuador	-	-	X	X	Approach: Quantitative Design: Multivariate analysis Population and Sample: 1676 university students from 11 universities in Ecuador Techniques and Instruments: Likert scale
6	( Wagner et al., 2024)	South Africa	-	X	X	X	Approach: Quantitative Design: Non-experimental Population and Sample: 596 first-year students surveyed between September and October 2020 Instrument: Questionnaire
7	( Castro-Martínez & Machuca-Téllez, 2023)	Colombia	X	X	X	X	Approach: Qualitative Design: Literature review Population and Sample: Not applicable Instrument: Document analysis
8	( Santos-Villalba et al., 2023)	Spain	X	X	X	X	Approach: Qualitative Design: Phenomenological Population and Sample: 12 students who had interrupted their studies Techniques and Instruments: Focus group discussions
9	( Llauró et al., 2023)	Spain	X	X	X	X	Approach: Mixed Design: Iterative and participatory Population and Sample: 1,742 students Instruments: Questionnaire and focus groups
10	( Schmidt et al., 2023)	Chile	X	X	X	X	Approach: Quantitative Design: Non-experimental, descriptive, and cross-sectional Population and Sample: 2222 university students, 2016 cohort, Universidad de La Frontera in Temuco, Chile (46% women and 54% men)
11	( Marte Espinal & Fabián V, 2021)	Dominican Republic	X	X	X	X	Design: Descriptive and non-experimental, with a cross-sectional design Population: 12,716 students from various programs at the Santiago Campus of UASD Sample: 415 students Instruments: Surveys and open interviews
12	( Guerrero & Espejo, 2024)	Colombia	X	X	X	X	Approach: Quantitative Design: Comparative-inferential Population and Sample: The sample comes from national databases such as SPADIES in Colombia and EducaBase in Spain, covering data from 2010 to 2022 Instrument: Questionnaire
13	( Kabashi et al., 2022)	Republic of Kosovo	-	X	X	X	Approach: Quantitative Design: Non-experimental Population and Sample: 650 respondents from FECE programs (14.75% of dropouts between the academic years 2011-2012 and 2015-2016) Techniques and Instruments: Questionnaire
14	( Candelario Navarrete et al., 2024)	Mexico	X	X	X	X	Approach: Mixed Design: Descriptive and interpretative Population and Sample: Data provided by the National Institute of Statistics and Geography (INEGI) through the National Survey of Occupation and Employment (ENOE) and by the National Council for the Evaluation of Social Development Policy (CONEVAL) Techniques and Instruments: Surveys and interviews
15	( Félix Ibarra et al., 2023)	Mexico	X	X	X	X	Approach: Qualitative Design: Descriptive Population and Sample: 19 out of 32 students from the 2017-2021 cohort of the Law and Social Sciences program at a public Mexican university who had abandoned their studies Techniques and Instruments: Interviews
16	( Garcés-Prettel et al., 2024)	Colombia	X	X	X	X	Approach: Quantitative Design: Cross-sectional Population and Sample: Convenience sample of 539 students Techniques and Instruments: Questionnaires
17	( Díaz-Barriga-Arceo et al., 2022)	Mexico	X	X	X	X	Approach: Mixed Design: Non-experimental Population and Sample: 268 students from the National Autonomous University of Mexico (UNAM) Techniques and Instruments: Content analysis.
18	( Lázaro Alvarez et al., 2020)	Cuba	X	X	X	X	Approach: Quantitative Design: Non-experimental Population: 1022 Cuban students Sample: 485 students Techniques and Instruments: Questionnaire
19	( Chalela-Naffah et al., 2020)	Colombia	X	X	X	X	Approach: Quantitative Design: Exploratory-descriptive Population and Sample: 3026 students (74.7% of the total population) Techniques and Instruments: Questionnaire
20	( González Sanzana & Arce Secul, 2021)	Chile	X	X	X	X	Approach: Quantitative Design: Non-experimental – descriptive Population and Sample: 784 Chilean students enrolled in teaching programs Techniques and Instruments: Questionnaire
21	( Garcés-Delgado et al., 2024)	Spain	X	X	X	X	Approach: Mixed Design: Multiple case study Population and Sample: 34 dropout students from three autonomous communities in Spain Techniques and Instruments: Interviews
22	( Valencia-Arias et al., 2023)	Colombia	X	X	X	X	Approach: Quantitative Design: Cross-sectional exploratory-descriptive Population and Sample: Higher education students in Colombia, sample of 372 students from a low-middle socioeconomic level Techniques and Instruments: Questionnaire
23	( Galve-González et al., 2023)	Spain	X	X	X	X	Approach: Quantitative Design: Descriptive non-experimental Population and Sample: 719 students from a public university in Spain, 521 were first-year students and 198 were second-year students Techniques and Instruments: Questionnaire

1 = Vocational Guidance (subcategory); 2 = Academic Performance (subcategory); 3 = Socioeconomic Status (subcategory); 4 = Institutional Aspect (subcategory).

**Table 2.** Research excluded at the last minute that apparently met the inclusion criteria.

No.	Authors, Year	Causes of exclusion
1	( Henriquez Cabezas & Vargas Escobar, 2022)	This research focused exclusively on developing an early warning system to prevent academic dropout by analyzing academic performance. Causes of dropout related to vocational guidance, academic performance, socioeconomic status, or institutional factors were not included.
2	( Zárate-Valderrama et al., 2021)	This study aimed to use classification models to identify patterns and predict potential cases of dropout among university students. Therefore, it did not prioritize the causes of student dropout in any of the subcategories addressed in this review.
3	( Hoyos Osorio & Daza Santacoloma, 2023)	This research, although presenting an important early warning system to identify first-semester students at high risk of dropout, does not comprehensively address the causes of dropout related to the categories and subcategories of this review.
4	( Castrillón-Gómez et al., 2020)	This interesting study focuses exclusively on predicting student dropout using data mining techniques. Therefore, its contributions are very useful for designing university strategies aimed at reducing student dropout. However, it is not fully aligned with the research question guiding this systematic review.
5	( Barradas Arenas & Cocón Juárez, 2022)	This research focused on evaluating the impact of a repository of e-learning tools and the didactic strategies applied to reduce failure rates in the Bachelor's program in Information Technology at the Autonomous University of Carmen. However, it does not prioritize other central subcategories in this review, such as vocational guidance, socioeconomic status, and institutional aspects.

### Information sources

The main databases used were Scopus, Web of Science, and Google Scholar. The search was conducted over a period of approximately 60 days, concluding in May 2024. During this time, the reference lists of identified studies were thoroughly reviewed to ensure no relevant sources were omitted.

### Search strategy

Descriptors such as University Dropout, Vocational Guidance, Academic Performance, Socio-Economic Status, and Institutional Aspect were used. This strategy allowed for a wide range of studies to be covered and ensured a comprehensive and representative data collection of the existing literature on the subject under study ( Table 3).

**Table 3.** Search strategies for all databases.

• TITLE (university AND dropout) AND PUBYEAR > 2019 AND (LIMIT-TO (DOCTYPE, "ar") AND (LIMIT-TO (OA, "all")) = 104 Results • (TITLE (university AND dropout) AND TITLE (vocational AND guidance)) AND PUBYEAR > 2019 = 17 Results • (TITLE (university AND dropout) AND TITLE (academic AND performance)) AND PUBYEAR > 2019 07 = 36 Results • TITLE (university AND dropout) AND TITLE (socio-economic AND status)) AND PUBYEAR > 2019 = 9 Results • (TITLE (university AND dropout) AND TITLE (institutional AND aspect)) AND PUBYEAR > 2019 = 14 Results

### Study selection

**Figure 1.**
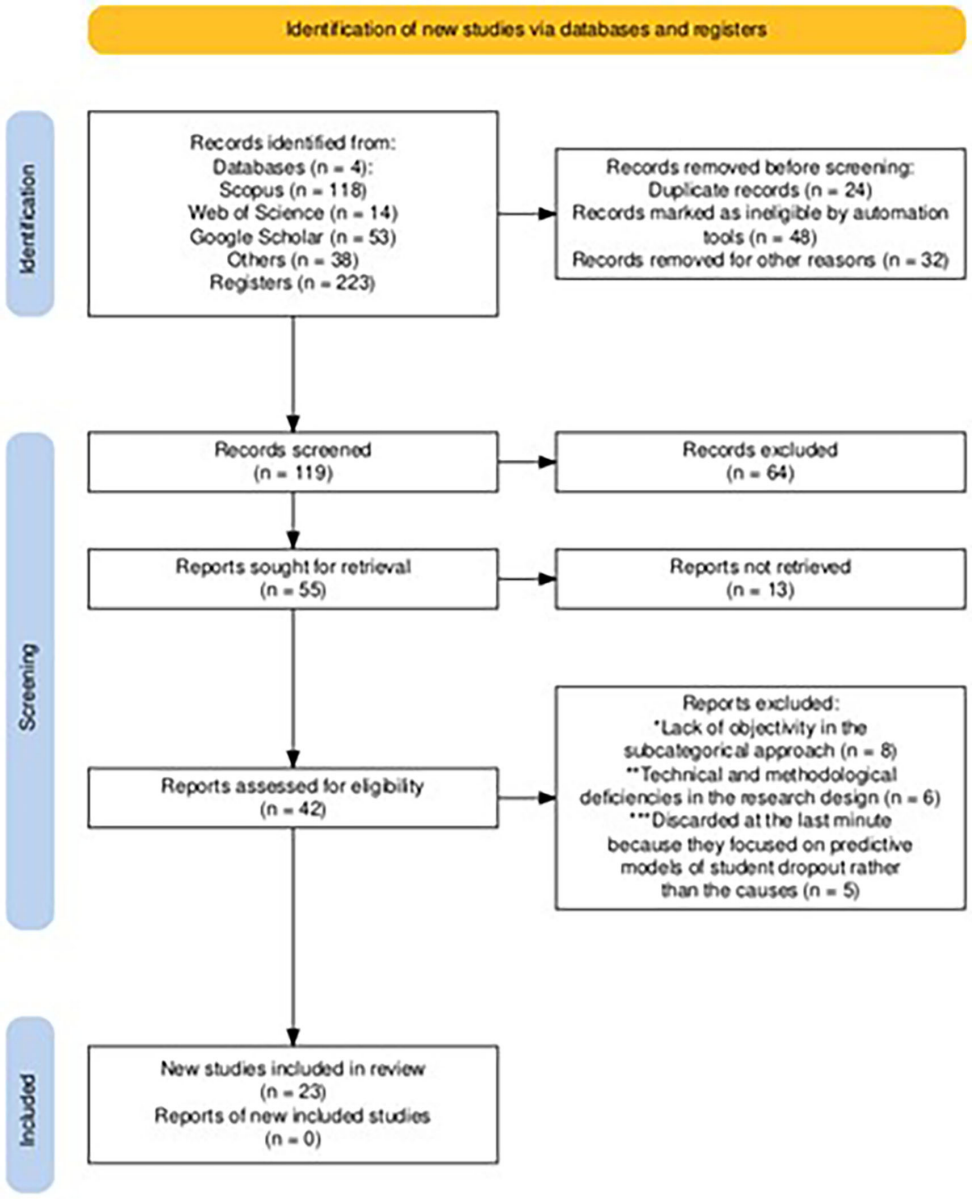
PRISMA flow diagram for article selection.

The process to determine if a study met the inclusion criteria was conducted in several stages. Two review authors independently screened each record and publication, reviewing titles and abstracts for preliminary selection. They then evaluated the full texts of the selected studies to confirm their eligibility. In cases of discrepancies, critical discussions were held to reach a consensus. Screening and inclusion were done manually, without the use of automation tools, to ensure accuracy and consistency in the application of the inclusion and exclusion criteria ( Figure 1).

### Data extraction

Two independent reviewers collected data from each publication, including details such as the article title, reference, year, study categories, approach, type of research, population and sample, and instruments used. The reviewers verified the data through discussions to resolve discrepancies, ensuring accuracy and consistency. No automation tools were used in this process.

### List of data

Data on university dropout were collected, focusing on the reasons and impact of dropout on students and institutions. Additionally, data were collected on participant characteristics (age, gender, socioeconomic level, and educational background) and intervention characteristics (type of programs or policies). Studies with incomplete, missing, or questionable information were excluded.

### Assessment of risk of bias in individual studies

To assess the risk of bias in qualitative studies, the criteria from the article “Validity criteria for qualitative research: three epistemological strands for the same purpose” ( Aráoz Cutipa & Pinto Tapia, 2021) were used, focusing on the credibility and transferability of the qualitative studies. For quantitative studies, the criteria from the article “Evaluating survey research in articles published in Library Science journals” ( Salvador-Oliván et al., 2021) were followed, evaluating survey design and data analysis. For mixed-method studies, both approaches were combined. Two authors independently conducted the assessment, resolving discrepancies by consensus.

### Methods of synthesis

To determine the eligibility of studies for synthesis, the characteristics of each study were tabulated and compared with the predefined inclusion criteria. A matrix was used, which included the article title, reference, year of publication, categories and subcategories, as well as relevant methodological information.

## Results

Table 2.2 in Extended data shows a total of 23 investigations, distributed as follows: 15 quantitative studies (65.22%), 3 qualitative studies (13.04%), and 5 mixed-method studies (21.74%). All studies (100%) addressed the subcategories of socioeconomic status and institutional aspect. The subcategory of academic performance was covered by 86% of the studies, while the subcategory of vocational guidance was addressed by 73.91% of the studies. The table also includes relevant general methodological information for each included study.

Within the issue of University Dropout, Vocational Guidance emerges as a determining factor in dropout rates. Various studies have indicated that the lack of adequate career guidance can lead students to make poor career choices, which often results in a disconnection from their studies and, ultimately, in dropout ( Castro-Martínez & Machuca-Téllez, 2023; Santos-Villalba et al., 2023; Thies & Falk, 2024). Thus, providing adequate guidance on study programs and professional training before students begin their studies allows them to make informed decisions, thereby reducing the risk of dropout ( Thies & Falk, 2024). Scientific evidence supports that vocational guidance not only helps students identify their interests and skills but also provides them with a clear vision of their future career paths, which can increase their motivation and commitment ( Candelario Navarrete et al., 2024; Castro-Martínez & Machuca-Téllez, 2023; Schmidt et al., 2023).

Many students abandon their studies upon realizing that the field they chose does not align with their personal interests or professional goals. This misalignment leads to a loss of motivation and can trigger identity and attachment crises ( Félix Ibarra et al., 2023; Santos-Villalba et al., 2023). Additionally, the lack of clear vocational guidance can make students feel disconnected and ill-prepared for the job market, increasing uncertainty about their professional future ( Candelario Navarrete et al., 2024; Garcés-Prettel et al., 2024).

Student motivation and interest in their study program significantly impact dropout rates. In this regard, students’ passion and commitment to their field of study are essential for preventing dropout ( Llauró et al., 2023). Conversely, poor career choices are a significant cause of dropout. A study included in this review found that 51.2% of students reported choosing the wrong profession ( Marte Espinal & Fabián V, 2021); therefore, the lack of adequate career guidance can also lead students to perceive their education as irrelevant to their future careers, resulting in early abandonment of their studies.

A study comparing university dropout rates between Spain and Colombia found that students’ disconnection from their studies, due to a lack of guidance, results in higher dropout rates ( Guerrero & Espejo, 2024). Other research indicates that insufficient vocational guidance can lead to a lack of commitment and, ultimately, to dropout ( Garcés-Prettel et al., 2024; Valencia-Arias et al., 2023). Additionally, the impact of social integration and family background also influences both students’ commitment to their studies and their decisions to drop out ( Lázaro Alvarez et al., 2020).

Academic Performance is also a critical and determining factor in university dropout. The studies included in this work show that students with higher grade point averages are at a lower risk of dropping out, both for native and international students; thus, it can be deduced that academic performance plays a crucial role in preventing dropout ( Thies & Falk, 2024). However, even students with high grade point averages, when facing emotional problems, cultural disconnection, lack of motivation, or even the perception of academic content as too simple and easy, can exhibit high dropout rates even in highly selective study programs ( Thomsen, 2022). This finding suggests that academic performance, although important, is not the only factor influencing student retention.

As previously mentioned, academic performance is not only related to grades but also to students’ self-concept in specific subjects. For example, a study found that self-concept in mathematics is positively related to academic achievement, which in turn reduces the likelihood of dropout ( Geisler et al., 2023). Conversely, low academic performance can lead to a lack of commitment and, ultimately, to dropout, especially when students are not diagnosed in time or do not receive adequate academic support ( Gonzales Lopez & Evaristo Chiyong, 2021).

Likewise, academic integration, such as strong bonds with peers and institutional commitment, is associated with a lower likelihood of dropout. Students who feel integrated into their academic environment are less likely to abandon their studies ( Wagner et al., 2024). However, when these students exhibit low academic performance, including learning difficulties and low grades, it becomes one of the main causes of student dropout. They may feel demotivated and decide to leave ( Castro-Martínez & Machuca-Téllez, 2023; Garcés-Delgado et al., 2024).

On the other hand, traditional teaching methods that fail to engage students can lead to feelings of failure and the decision to drop out of university ( Santos-Villalba et al., 2023). It is also worth considering study habits and the amount of time students dedicate to this activity; as is known, nonexistent, inadequate, and ineffective study habits are correlated with low academic performance, which in turn leads to higher student dropout rates ( Candelario Navarrete et al., 2024; Llauró et al., 2023). This emphasizes the need and importance of including pedagogical techniques in study programs that foster engagement and active participation of students in their learning process. Otherwise, low academic performance, lack of understanding of content, and even failing exams could be causes of school dropout ( Guerrero & Espejo, 2024; Kabashi et al., 2022).

Regarding Socioeconomic Status, financial problems stand out as a significant cause of school dropout, as a better financial situation is related to a higher probability of academic success and a lower risk of dropout ( Thies & Falk, 2024). In this context, economically disadvantaged students are about 15 percentage points more likely to drop out compared to their peers with better economic conditions, even after accounting for other factors such as grades in previous educational stages ( Thomsen, 2022).

Most of the studies included in this research suggest that economic difficulties are one of the main causes of school dropout. Under this premise, students often leave school due to financial constraints, the need to work, or family responsibilities ( Candelario Navarrete et al., 2024; Gonzales Lopez & Evaristo Chiyong, 2021). This situation must be observed with caution because employment opportunities during the early years of study can distract students from their academic responsibilities, making it difficult for them to interact with their peers and professors, thereby increasing dropout rates ( Kabashi et al., 2022). Therefore, factors such as financial pressures and the eventual need for some students to economically support their families are critical causes affecting student retention ( Guerrero & Espejo, 2024).

On the other hand, the lack of technical and technological resources necessary for online learning also influences the decision to drop out. This situation gained significant momentum with the onset of the COVID-19 pandemic, where many students worldwide were forced to leave their studies for this reason ( Pertegal-Felices et al., 2022). Additionally, students from lower socioeconomic backgrounds, especially those facing food insecurity, are also prone to dropping out. When these students face severe food insecurity, their likelihood of dropping out more than doubles, making these factors key contributors to increasing dropout rates ( Wagner et al., 2024). Furthermore, limited access to scholarships and financial support also influences school dropout rates. Students from lower socioeconomic backgrounds or those who do not receive adequate financial support for their studies are at a higher risk of dropping out ( Llauró et al., 2023; Marte Espinal & Fabián V, 2021).

Regarding Institutional Aspects, satisfaction with study programs, integration into the academic environment, and the good reputation of the university are critical factors that reduce the likelihood of school dropout, especially among international students ( Thies & Falk, 2024). In Denmark, for example, the lack of elite universities leads the most advantaged students to seek out specific prestigious programs, thereby increasing dropout rates. This is because social selectivity depends more on a specific study program than on the perception of any particular institution ( Thomsen, 2022).

Additionally, the transition from regular basic education to higher education, as well as the nature of university programs (especially when they do not meet students’ expectations), can significantly influence dropout rates. For example, the difference in cognitive demands between school and university mathematics can lead to dissatisfaction and dropout ( Geisler et al., 2023). Therefore, a positive transition to university life and effective tutoring actions can significantly reduce the likelihood of students dropping out ( Llauró et al., 2023). Other institutional factors such as the quality of support services, modular design, and faculty guidance also have a significant impact on dropout rates. Effective institutional support and well-designed curricular experiences can help reduce dropout rates by keeping students engaged and supported ( Gonzales Lopez & Evaristo Chiyong, 2021).

Similarly, educational institutions, especially those of higher education, must develop better resilience strategies and support networks to help students cope with emerging crises such as the COVID-19 pandemic ( Pertegal-Felices et al., 2022). During this period, the lack of institutional adaptation, coupled with outdated technological infrastructures, spiked student dropout rates. Additionally, factors such as class duration and ineffective or inadequate student tutoring systems were associated with higher dropout rates, highlighting the need for better institutional support ( Wagner et al., 2024).

On the other hand, inflexible curricula and insufficient academic advising can lead to school dropout, as students may feel unsupported and unable to balance their studies with other responsibilities ( Castro-Martínez & Machuca-Téllez, 2023). The results of this review also indicate that the quality of education, the lack of commitment from educators, and outdated educational policies contribute to exacerbating dropout rates ( Santos-Villalba et al., 2023).

Finally, institutions that do not provide a conducive and engaging environment contribute to higher dropout rates ( Guerrero & Espejo, 2024). Likewise, those with rigid or inflexible institutional policies can also lead students to abandon their studies ( Candelario Navarrete et al., 2024; Félix Ibarra et al., 2023; Kabashi et al., 2022). Therefore, collaboration between directors, administrators, professors, and students is essential to effectively address the issue of university dropout ( Galve-González et al., 2023; Garcés-Prettel et al., 2024; González Sanzana & Arce Secul, 2021; Valencia-Arias et al., 2023).

## Conclusions

Vocational Guidance plays a crucial role in reducing university dropout rates. The contributions of these researchers demonstrate that providing adequate career guidance can help students make informed decisions, aligning their interests and abilities with their professional goals, which in turn increases their motivation and commitment to their studies.

Academic Performance is a crucial factor in preventing university dropout. Scientific evidence indicates that students with better grades have a lower risk of abandoning their studies. However, additional factors such as academic support, integration into the university community, and socioeconomic challenges also play a significant role. Addressing these areas through targeted interventions can improve student retention and reduce dropout rates.

Regarding Socioeconomic Status, factors such as family socioeconomic level and economic pressure significantly influence dropout rates. Economic challenges are a common reason why students leave their studies. Research included in this investigation has shown that there is a correlation between financial aid and dropout rates, as students who receive financial assistance are less likely to abandon their studies, especially at the beginning of their university education.

Finally, Institutional Aspects are fundamental in preventing university dropout. Factors such as the quality of support services, social and academic integration, and appropriate educational policies can significantly improve student retention. Scientific evidence suggests that a comprehensive strategy that includes strong institutional support and mentoring programs can reduce dropout rates and ensure students’ academic success.

### Registration and protocol

PROSPERO: ID: 565816, Title: University Dropout: A Systematic Review of the Main Determinant Factors.

## Data Availability

All data underlying the results are available as part of the article and no additional source data are required. **Reporting guidelines** Zenodo: University dropout: A systematic review of the main determinant factors. https://doi.org/10.5281/zenodo.13117695 ( Quincho Apumayta et al., 2024). This project contains the following underlying data:
• 01 PRISMA_2020_checklist.pdf • 02 Search strategies for all databases, and main results.pdf Data are available under the terms of the Creative Commons Attribution 4.0 International license (CC-BY 4.0).

## References

[ref1] Aráoz Cutipa RA Pinto Tapia B : Validity criteria for qualitative research: three epistemological strands for the same purpose. *Summa Psicológica UST.* 2021;18(1):47–56. 10.18774/0719-448x.2021.18.485

[ref2] Barradas Arenas UD Cocón Juárez JF : Learning tools as a means of support in the reduction of failure at the universitaria autonoma del carmen for the distance modality. *Revista Iberoamericana Para La Investigación y El Desarrollo Educativo.* 2022;13(25):e419. 10.23913/ride.v13i25.1340

[ref3] Bernardo A Cervero A Esteban M : Freshmen program withdrawal: Types and recommendations. *Front. Psychol.* 2017;8:1–11. 10.3389/fpsyg.2017.01544 28983263 PMC5613773

[ref37] Brennan S Munn Z : PRISMA 2020: A reporting guideline for the next generation of systematic reviews. *JBI Evid. Synth.* 2021;19(5):906–908. 10.11124/JBIES-21-00112 33989266

[ref4] Candelario Navarrete F Ávila Romero R Juárez Olascoaga BG : Analysis of poverty as a factor in the dropout rate of university students in Mexico City from 2000 to 2022. *Salud, Ciencia y Tecnología - Serie de Conferencias.* 2024;3:738. 10.56294/sctconf2024738

[ref5] Castrillón-Gómez OD Sarache W Ruiz-Herrera S : Prediction of main variables that lead to student dropout by using data mining techniques. *Formacion Universitaria.* 2020;13(6):217–228. 10.4067/S0718-50062020000600217

[ref6] Castro-Martínez JA Machuca-Téllez G : University dropout in Latin America: An ecological perspective. *Estudios Pedagógicos (Valdivia).* 2023;49(2):87–108. 10.4067/S0718-07052023000200087

[ref7] Chalela-Naffah S Valencia-Arias A Ruiz-Rojas GA : Psycho-social and familial factors influencing drop-out rates among university students in the context of developing countries. *Revista Lasallista de Investigacion.* 2020;17(1):103–115. 10.22507/rli.v17n1a9

[ref10] Félix Ibarra AV Urrea Zazueta ML López Leyva S : School dropout of university students in the career of Law and Social Sciences. *Revista de Ciencias Sociales.* 2023;XXIX(2):242–254. 10.31876/rcs.v29i2.39974

[ref11] Galve-González C Bernardo AB Castro-López A : Understanding the dynamics of college transitions between courses: Uncertainty associated with the decision to drop out studies among first and second year students. *European Journal of Psychology of Education.* 2023;39:959–978. 10.1007/s10212-023-00732-2

[ref12] Garcés-Delgado Y Fernández-Esteban MI Álvarez-Pérez PR : The process of adaptation to higher education studies and its relation to academic dropout. *European Journal of Education.* 2024; (e12650):1–20. 10.1111/ejed.12650

[ref13] Garcés-Prettel M De la Ossa-Robinson S Arellano-Cartagena W : Return or Not to Return to In-Person Classes? Motivations and Fears that Influence University Dropout in Colombia in the Post-Pandemic Era. *Salud Uninorte.* 2024;40(1):52–68. 10.14482/sun.40.01.159.456

[ref14] Geisler S Rach S Rolka K : The relation between attitudes towards mathematics and dropout from university mathematics—the mediating role of satisfaction and achievement. *Educational Studies in Mathematics.* 2023;112:359–381. 10.1007/s10649-022-10198-6

[ref16] González Sanzana Á Arce Secul R : Personal and access factors that affect the retention and dropout of pre-service teachers at a chilean university in an extreme geographic area. *SOPHIA AUSTRAL.* 2021;27(1):1–19. 10.22352/SAUSTRAL202127001

[ref15] Gonzales Lopez E Evaristo Chiyong I : Academic Achievement and Dropout of University Students from a Course in Both an Online and Face-to-Face Modality. *RIED-Revista Iberoamericana de Educacion a Distancia.* 2021;24(2):189–202. 10.5944/ried.24.2.29103

[ref17] Guerrero SC Espejo RL : Deserción universitaria: estudio comparativo entre Colombia y España desde la perspectiva de género. *Formación Universitaria.* 2024;17(2):101–112. 10.4067/S0718-50062024000200101

[ref38] Guzmán Rincón A Barragán Moreno S Cala-Vitery F : Rural Population and COVID-19: A Model for Assessing the Economic Effects of Drop-Out in Higher Education. Front. Educ. 2021;6. 10.3389/feduc.2021.812114

[ref18] Henriquez Cabezas N Vargas Escobar D : Predictive models of academic achievement and dropout of first year students of a chilean public university. *Revista de Estudios y Experiencias En Educación.* 2022;21(45):299–316. 10.21703/0718-5162.v21.n45.2022.015

[ref19] Hoyos Osorio JK Daza Santacoloma G : Predictive Model to Identify College Students with High Dropout Rates. *Revista Electronica de Investigacion Educativa.* 2023;25(e13):1–10. 10.24320/REDIE.2023.25.E13.5398

[ref20] Kabashi Q Shabani I Caka N : Analysis of the Student Dropout Rate at the Faculty of Electrical and Computer Engineering of the University of Prishtina, Kosovo, from 2001 to 2015. *IEEE Access.* 2022;10:68126–68137. 10.1109/ACCESS.2022.3185620

[ref21] Kim D Kim S : Sustainable education: Analyzing the determinants of university student dropout by nonlinear panel data models. *Sustainability.* 2018;10(4):1–18. 10.3390/su10040954

[ref22] Lázaro Alvarez N Callejas Z Griol D : Factors that affect student desertion in careers in Computer Engineering profile. *Revista Fuentes.* 2020;22(1):105–126. 10.12795/revistafuentes.2020.v22.i1.09

[ref23] Llauró A Fonseca D Romero S : Identification and comparison of the main variables affecting early university dropout rates according to knowledge area and institution. *Heliyon.* 2023;9(6):e17435. 10.1016/j.heliyon.2023.e17435 37441382 PMC10333612

[ref39] Lorenzo-Quiles O Galdón-López S Lendínez-Turón A : Dropout at university: Variables involved on it. Front. Educ. 2023;8. 10.3389/feduc.2023.1159864

[ref24] Marte Espinal R Fabián VL : Determinants of university dropout: a case study in the Dominican Republic. *Sapienza: Int. J. Interdiscip. Stud.* 2021;2(1):255–268. 10.51798/sijis.v2i1.76

[ref40] Mtshweni BV : Adjustment and socioeconomic status: How do these factors influence the intention to dropout of university? S. Afr. J. Psychol. 2021;52(3):262–274. 10.1177/00812463211059141

[ref41] OECD : *Education at a Glance 2019: OECD Indicators.* OECD Publishing;2019. 10.1787/f8d7880d-en

[ref25] Page MJ McKenzie JE Bossuyt PM : Declaración PRISMA 2020: una guía actualizada para la publicación de revisiones sistemáticas. *Rev. Esp. Cardiol.* 2021;74(9):790–799. 10.1016/J.RECESP.2021.06.016 34446261

[ref26] Pertegal-Felices ML Valdivieso-Salazar DA Espín-León A : Resilience and Academic Dropout in Ecuadorian University Students during COVID-19. *Sustainability.* 2022;14:1–12. 10.3390/su14138066

[ref27] Quincho Apumayta R Carrillo Cayllahua J Ccencho Pari A : University dropout: A systematic review of the main determinant factors (Version 1). Zenodo.[Dataset].2024. 10.5281/ZENODO.13117695 PMC11561383 39544919

[ref28] Salvador-Oliván JA Marco-Cuenca G Arquero-Avilés R : Evaluating survey research in articles published in Library Science journals. *Revista Espanola de Documentacion Cientifica.* 2021;44(2):1–18. 10.3989/redc.2021.2.1774

[ref9] Santos RSS dos Ponti MA Hora Rodrigues KR da : The Use of Digital Reports to Support the Visualization and Identification of University Dropout Data. *Lecture Notes in Computer Science (Including Subseries Lecture Notes in Artificial Intelligence and Lecture Notes in Bioinformatics).* 2022;13305. 10.1007/978-3-031-06424-1_23

[ref29] Santos-Villalba MJ Olmo A del Fernández MJ : Incident factors in Andalusian university dropout: A qualitative approach from the perspective of higher education students. *Frontiers in Education.* 2023;7:1–14. 10.3389/feduc.2022.1083773

[ref30] Schmidt BJ Boero P Méndez Vera JA : Factors influencing university dropouts: The case of a Chilean state university. * Revista Portuguesa De Educação,* 2023;36(1):e23002. 10.21814/rpe.23401

[ref31] Thies T Falk S : Which Factors Drive Major Change and University Dropout? An Analysis on International Degree-Seeking Students at German Universities. *Journal of International Students.* 2024;15(1):326–346. 10.32674/jis.v15i1.5434

[ref32] Thomsen JP : The social class gap in bachelor’s and master’s completion: university dropout in times of educational expansion. *Higher Education.* 2022;83(5):1021–1038. 10.1007/s10734-021-00726-3

[ref33] Valencia-Arias A Chalela S Cadavid-Orrego M : University Dropout Model for Developing Countries: A Colombian Context Approach. *Behavioral Sciences.* 2023;13(5):1–14. 10.3390/bs13050382 37232619 PMC10215226

[ref34] Viloria A Garcia Padilla J Vargas-Mercado C : Integration of Data Technology for Analyzing University Dropout. *Procedia Computer Science.* 2019;155:569–574. 10.1016/j.procs.2019.08.079

[ref35] Wagner F Wagner RG Makuapane LP : Mental distress, food insecurity and university student dropout during the COVID-19 pandemic in 2020: evidence from South Africa. *Frontiers in Psychology.* 2024;15(February):1–10. 10.3389/fpsyt.2024.1336538 38380123 PMC10876832

[ref36] Zárate-Valderrama J Bedregal-Alpaca N Cornejo-Aparicio V : Classification models to recognize patterns of desertion in university students Joshua. *Ingeniare. Revista Chilena de Ingeniería.* 2021;29(1):168–177. 10.4067/S0718-33052021000100168

