# Supplementary material for: University Dropout: A Systematic Review of the Main Determinant Factors (2020-2024)
Source: F1000Res. 2024 Nov 12;13:942. Originally published 2024 Aug 20. [Version 2] doi: 10.12688/f1000research.154263.2 (PMC11561383; doi:10.12688/f1000research.154263.2)
Supplement: Peer review history

# Reviewer report: Reviewer response for version 2

**DOI:** 10.5256/f1000research.174226.r339842

**Published:** 2024-11-13

**Authors:** Enaidy Reynosa Navarro

**Affiliations:** 1 Universidad Cesar Vallejo, Trujillo, La Libertad, Peru

I have no further comments to make. I am fully satisfied with the revisions made by the authors following the completion of the first round of review. The improvements have addressed all previous concerns effectively

Are the rationale for, and objectives of, the Systematic Review clearly stated?

Yes

Is the statistical analysis and its interpretation appropriate?

Not applicable

If this is a Living Systematic Review, is the ‘living’ method appropriate and is the search schedule clearly defined and justified? (‘Living Systematic Review’ or a variation of this term should be included in the title.)

Not applicable

Are sufficient details of the methods and analysis provided to allow replication by others?

Yes

Are the conclusions drawn adequately supported by the results presented in the review?

Yes

Reviewer Expertise:

Education and Educational Quality, Quality Education, and Education Administration

I confirm that I have read this submission and believe that I have an appropriate level of expertise to confirm that it is of an acceptable scientific standard.

# Reviewer report: Reviewer response for version 2

**DOI:** 10.5256/f1000research.174226.r339843

**Published:** 2024-11-13

**Authors:** Nicole Caldichoury-Obando

**Affiliations:** 1 Universidad de Los Lagos, Osorno, Los Lagos Region, Chile

The current version of the article has incorporated the suggestions, therefore, it has clarified several aspects, being approved.

Are the rationale for, and objectives of, the Systematic Review clearly stated?

Yes

Is the statistical analysis and its interpretation appropriate?

Yes

If this is a Living Systematic Review, is the ‘living’ method appropriate and is the search schedule clearly defined and justified? (‘Living Systematic Review’ or a variation of this term should be included in the title.)

Not applicable

Are sufficient details of the methods and analysis provided to allow replication by others?

Partly

Are the conclusions drawn adequately supported by the results presented in the review?

Partly

Reviewer Expertise:

Psychologist, Master's Degree in Education with a Mention in Educational Guidance. Neurosciences applied to education. Neurodevelopmental disorders and special education.

I confirm that I have read this submission and believe that I have an appropriate level of expertise to confirm that it is of an acceptable scientific standard.

# Reviewer report: Reviewer response for version 1

**DOI:** 10.5256/f1000research.169265.r317129

**Published:** 2024-09-30

**Authors:** Nicole Caldichoury-Obando

**Affiliations:** 1 Universidad de Los Lagos, Osorno, Los Lagos Region, Chile

The objective of this article is to systematize the scientific evidence on the causes of university dropout, focusing on the subcategories of vocational orientation, academic performance, socioeconomic level, and institutional aspects between the years 2020-2024.

 Regarding the method, it is not clearly defined, but it is inferred that it is of the theoretical or bibliographic research type with primary sources of information, where scientific articles published in indexed journals were used.

 The main conclusions are related to the relevance of generating vocational guidance processes to reverse university dropout, along with considering early warnings regarding university academic performance, since it is considered a good predictor of the successful university trajectory, it is also necessary to be able to count on academic and financial support to achieve greater integration and adherence to university life.

 In general terms, the article is clear and consistent with respect to the title, objectives, base question and keywords.  It has a structure that contributes to the achievement of the objective and the proposed results, where updated bibliographic sources are cited, reaching logical conclusions. However, certain methodological procedures must be clarified in order to be able to replicate the study and provide foundations for the relevance of the study. Below are the details and topics to be improved:

*1.- Title*

 Strengths (S):  Clear and precise.

 Limitation (L): As a suggestion add the years 2020-2024.

*2.- Summary*

 S:  It provides a synthesis of the causes of university dropout between 2020-2024 in categories and subcategories, informs the number of selected scientific articles.

 L: Clarify and state the scientific method used and the search techniques used.

*3.-Introduction*

 S: Context and review with references to studies. They mention university dropout as a multifaceted problem in which several factors influence. Inadequate vocational guidance, low academic performance, along with socioeconomic level as critical determinants, are alluded to, in addition to institutional aspects such as the academic environment and institutional support, motivation, self-esteem and frustration are also included. The basic question and objective are clear.

 L: It is important to mention all the key factors of university dropout, accompanied by their respective citations, as part of the justification.

 Clearly explain why it is relevant to study university dropouts, providing figures such as the number of students affected, and/or the amount of money involved.

 School dropout vs. university dropout, only focus on a standard term, since they are different age ranges and it is confusing.

*4.- Methodology*

 S: They declare a method with inclusion and exclusion criteria, in addition to the sources of information and the time spent, tables are provided with search strategies and data extraction. Regarding the synthesis method, they mention a data matrix with the inclusion criteria, title, reference, year of publication, categories, subcategories, and the method used.

 L: The method indicates the title of an article and its author (Page et al., 2021), but does not declare the method. After reviewing the cited article, it is clear that they are guided by PRISMA 2020 criteria, but it is necessary to declare it.

 Within the inclusion criteria, concerns arise regarding whether they considered articles only in Spanish, English or in all languages, the studies were carried out in public or private universities, with what number of students, and the results of the studies reviewed.

 Regarding the selection of the studies, where Table 1 is attached, they must mention these data, refer to the criteria on which they worked to clean the data. And mention how many articles were excluded and why.

 In the data extraction, they do not mention the results of the studies reviewed, which they refer to later in the results section.

 In a list of data, they declare them, but it is necessary to provide a table.

 Risk of bias assessment in individual studies, cites the title of 2 articles and their authors (Aráoz Cutipa & Pinto Tapia, 2021; Salvador-Oliván et al., 2021), it is considered pertinent to be precise and indicate the criteria.

 Regarding the synthesis method, they mention a data matrix, but how many subjects participated in the studies (quantitative and qualitative), what techniques they worked with, the gender of the participants, country, language, results.

*5.- Results*

 S: Finally, they mention the selection of 23 studies (15 quantitative, 3 qualitative and 5 mixed) according to their search criteria, where all meet the categories of socioeconomic level and institutional aspect and 86% with academic performance and 73.91% with vocational orientation.  Data are provided on other interesting components of these categorizations such as motivation, commitment, bonds with peers, self-concept, learning difficulties, study habits, teaching methods, technological resources, among others.

 L: Include the table with the data of the 23 studies reviewed in the declared criteria, including their results and conclusions.

*6.- Conclusion*

 S: Importance of focusing on vocational guidance in order to avoid university dropout. Academic performance, socioeconomic level, and institutional aspects are linked as relevant elements to prevent university dropout.

 It has registries and protocols, as well as underlying data.

 L: Use the term dropout rather than abandonment for internal coherence, if they refer to the same thing, otherwise clarify it.

 What was the limitation of the study, indicate.

 The study was funded.

 There are or are not conflicts of interest.

*Conclusion of the article*: It has a logical structure that they develop, but they must improve the points indicated, completing and/or clarifying information, the delivery of tables with the data will help to clear doubts and will deliver value to their work for its replicability.

*Approval status*: Approved with reservations. The article has sufficient merits for indexing, as explained, it is coherent in the topics it wishes to explore. However, certain adjustments are required to enhance what has been done.

Are the rationale for, and objectives of, the Systematic Review clearly stated?

Yes

Is the statistical analysis and its interpretation appropriate?

Yes

If this is a Living Systematic Review, is the ‘living’ method appropriate and is the search schedule clearly defined and justified? (‘Living Systematic Review’ or a variation of this term should be included in the title.)

Not applicable

Are sufficient details of the methods and analysis provided to allow replication by others?

Partly

Are the conclusions drawn adequately supported by the results presented in the review?

Partly

Reviewer Expertise:

Psychologist, Master's Degree in Education with a Mention in Educational Guidance. Neurosciences applied to education. Neurodevelopmental disorders and special education.

I confirm that I have read this submission and believe that I have an appropriate level of expertise to confirm that it is of an acceptable scientific standard, however I have significant reservations, as outlined above.

## References

[1] : University Dropout: A Systematic Review of the Main Determinant Factors. *F1000Research* .2024;13: 10.12688/f1000research.154263.1 10.12688/f1000research.154263.1 PMC1156138339544919

# Reviewer report: Reviewer response for version 1

**DOI:** 10.5256/f1000research.169265.r317134

**Published:** 2024-09-12

**Authors:** Enaidy Reynosa Navarro

**Affiliations:** 1 Universidad Cesar Vallejo, Trujillo, La Libertad, Peru

The rationale and objectives are clearly stated. This systematic review focuses on understanding the causes of university dropout, with an emphasis on vocational guidance, academic performance, socioeconomic status, and institutional factors during the period from 2020 to June 2024.

 The review also provides sufficient details on the methods and analysis, enabling replication by other researchers. The inclusion criteria are clearly specified (studies published between 2020 and 2024, standardized methodological approaches), as well as the databases used (Scopus, Web of Science, Google Scholar) and the search strategy (using specific descriptors such as "university dropout" and related terms). Additionally, the study selection process is detailed, including independent review by two authors and manual data extraction to ensure accuracy. An interesting aspect is that, to assess the risk of bias in qualitative studies, the validity criteria for qualitative research proposed by experts (Aráoz Cutipa & Pinto Tapia, 2021) were used. For quantitative studies, the criteria proposed in the research “Assessment of Survey-Based Research in Articles Published in Library Science Journals” (Salvador-Oliván et al., 2021) were followed, evaluating survey design and data analysis. For mixed-method studies, both approaches were combined. All these steps ensure that the study can be effectively replicated.

 Statistical analysis was not conducted in this systematic review because, being based on the PRISMA protocol, its main focus was the collection, evaluation, and synthesis of existing studies, rather than a meta-analysis, which would require direct statistical analysis. The applied PRISMA protocol focused on ensuring transparency and comprehensiveness in the literature review through rigorous study selection, risk of bias assessment, and qualitative synthesis of findings, providing essential information for preventing university dropout.

 The conclusions are clearly supported by the review's findings. The research demonstrated, through the analysis of 23 studies, that vocational guidance, academic performance, socioeconomic status, and institutional factors are critical determinants in university dropout. These conclusions reflect a coherent synthesis of the findings, highlighting the need to implement specific interventions in these areas. It is recommended that educational institutions strengthen these aspects, providing adequate vocational guidance and financial support to significantly reduce student dropout and improve academic retention.

Are the rationale for, and objectives of, the Systematic Review clearly stated?

Yes

Is the statistical analysis and its interpretation appropriate?

Not applicable

If this is a Living Systematic Review, is the ‘living’ method appropriate and is the search schedule clearly defined and justified? (‘Living Systematic Review’ or a variation of this term should be included in the title.)

Not applicable

Are sufficient details of the methods and analysis provided to allow replication by others?

Yes

Are the conclusions drawn adequately supported by the results presented in the review?

Yes

Reviewer Expertise:

Education and Educational Quality, Quality Education, and Education Administration

I confirm that I have read this submission and believe that I have an appropriate level of expertise to confirm that it is of an acceptable scientific standard.

## References

[1] : Evaluación de la investigación con encuestas en artículos publicados en revistas del área de Biblioteconomía y Documentación. *Revista Española de Documentación Científica* .2021;44(2) : 10.3989/redc.2021.2.1774 10.3989/redc.2021.2.1774

[2] : Validity criteria for qualitative research: three epistemological strands for the same purpose. Summa Psicológica UST.
